# Effectiveness of corticosteroid injections in adhesive capsulitis of shoulder: A meta-analysis

**DOI:** 10.1097/MD.0000000000007529

**Published:** 2017-07-14

**Authors:** Wei Wang, Mingmin Shi, Chenhe Zhou, Zhongli Shi, Xunzi Cai, Tiao Lin, Shigui Yan

**Affiliations:** Department of Orthopedic Surgery, Second Affiliated Hospital, School of Medicine, Zhejiang University, Hangzhou, China.

**Keywords:** adhesive capsulitis of the shoulder, corticosteroid injection, pain, range of motion

## Abstract

**Background:**

Primary adhesive capsulitis is mainly characterized by spontaneous chronic shoulder pain and the gradual loss of shoulder motion. The main treatment for adhesive capsulitis is a trial of conservative therapies, including analgesia, exercise, physiotherapy, oral nonsteroidal anti-inflammation drugs, and intra-articular corticosteroid injections. Previously, it was reported that intra-articular corticosteroid lead to fast pain relief and improvement of range of motion (ROM). The objective of this study was to determine whether corticosteroid injections would lead to better pain relief and greater improvement in ROM.

**Methods:**

We searched PubMed, Medline, and the Cochrane library. We included 5 articles of the 1166 articles identified. Totally injection group included 115 patients and placebo group included 110 patients. We calculated the weighted mean differences to evaluate the pain relief as the primary outcome. We determined the ROM as the secondary outcome. Study quality was evaluated using the 12-item scale. We also used the criteria of the Grading of Recommendations Assessment, Development and Evaluation to evaluate the quality of evidence.

**Results:**

In total, 5 studies were included, 4 of which were randomized clinical trials, with a sample size of 225 patients with adhesive capsulitis of the shoulders. The overall pooled data demonstrated that, compared with placebo as control treatment, intra-articular corticosteroid injections were more effective in reducing the pain score at 0 to 8 weeks, but there was no difference between the injection group and the control group at 9 to 24 weeks. Improvement of ROM in the injection group was greater than that of the control group both at 0 to 8 and 9 to 24 weeks.

**Conclusions:**

Intra-articular corticosteroid injections were more effective in pain relief in the short term, but this pain relief did not sustain in the long term. Intra-articular corticosteroid injection resulted in greater improvement in passive ROM both in the short and the long terms.

## 1 Introduction

Primary adhesive capsulitis of the shoulder (ACS), or “frozen shoulder,” is an inflammation of articular capsule which is usually aseptic. It was first introduced by Duplay in 1896.^[1]^ It is mainly characterized by spontaneous chronic shoulder pain and gradual loss of shoulder motion including all active and passive movements.^[2]^ The pathogenesis of primary adhesive capsulitis remains unclear.^[3]^ Patients with adhesive capsulitis first encounter a phase of “freezing” when increasing pain and stiffness last for several months, followed by a steady-state stage of “frozen” when shoulder motion is lost, then progressing into a “thawing” phase which presents less pain and return of the restricted motion.^[4,5]^ Although adhesive capsulitis is thought to be self-limited, complete resolution of the pain and disability does not always occur. Only 59% of the patients regain normal function after 4 years.^[6]^ The main treatment for adhesive capsulitis is a trial of conservative therapies, including analgesia, exercise, physiotherapy, oral nonsteroidal anti-inflammation drugs (NSAIDs), and intra-articular corticosteroid injections.^[7]^ It was previously reported that intra-articular corticosteroids lead to fast pain relief and improvement of range of motion (ROM).^[8–12]^

Buchbinder et al^[13]^ performed a systematic review of randomized and pseudo-randomized trials of corticosteroid injections for shoulder pain, and their conclusion was that corticosteroid injections may be effective. Griesser et al^[14]^ performed a systematic review of randomized-controlled trials (RCTs) and concluded that intra-articular corticosteroid injections lead to greater improvements in pain relief and ROM both in the short and the long terms, but compared to other treatments, the effects were similar in the long term. Sun et al have comparing Steroid injection with Nonsteroidal Anti-inflammatory Agents (NSAIDs)^[15]^ and physiotherapy^[16]^ for shoulder pain and concluded that steroid injection and physiotherapy were equally effective for patients with ACS and provided slightly more improvement in shoulder function without superiority in pain relief or risk of complications at 4 to 6 weeks comparing with NSAIDs. However, the effects of intra-articular injections comparing with placebo for ACS remained unclear. Therefore, we performed a meta-analysis comparing patients with adhesive capsulitis treated with intra-articular injections of corticosteroid and placebo to determine whether corticosteroid injections would lead to better pain relief and greater improvement in ROM. We also asked whether the efficacy of corticosteroid injections is different in the short and long terms.

## 2 Materials and methods

### 2.1 Study design

A meta-analysis and systematic review was conducted according to predefined guidelines provided by Cochrane Collaboration (2008) as described previously.^[15,16]^ All data were reported according to the Quality of Reporting for Meta-analysis provided by the Handbook for Systematic Reviews of Intervention Version 5.3.^[17]^

### 2.2 Literature research

**Figure 1:**
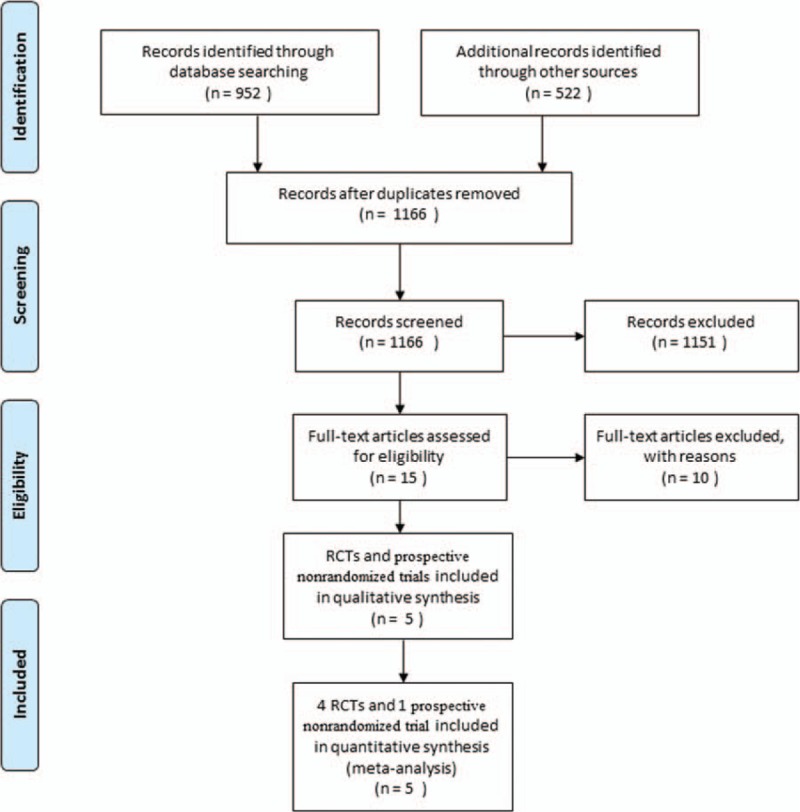
Flowchart illustrating the selection of the 5 trials included in our meta-analysis.

We searched Electronic databases (PubMed, Web of Science, and the Cochrane library) with the following keywords: Duplay disease, bursitis, capsulitis, frozen shoulder, stiff shoulder, periarthritis, intra-articular injection, and corticosteroid. All the databases were searched by 2 independent investigators (MS and WW), which was last updated on April 25, 2016. Reference lists of all the selected articles were hand-searched for any additional trials (Fig. 1). All analyses were based on previous published studies. Therefore, no ethical approval and patient consent are required.

### 2.3 Inclusion and exclusion criteria

The literature search initially yielded 1166 relevant trials, which is combined from PubMed (N = 952), Web of Science (N = 473), and the Cochrane library (N = 49) with 308 duplicates excluded. Two of us (ZS and SY) reviewed the titles and abstracts of all 1166 trials. We included those in which the target population consisted of patients undergoing primary adhesive capsulitis, the intervention was intra-articular injection of corticosteroid, the control procedure was sham injection, oral medications or no procedure, the outcomes included pain score and ROM, and the trial was an RCT or prospective, nonrandomized, controlled trials. Trials were excluded if they were Phase I or observational studies, case reports, or reviews, both data of pain score and ROM were unavailable, and the RCTs had a follow-up <2 weeks. By reading the titles and abstracts of these 1166 studies, we excluded 1151 because they did not fulfill the selection criteria. By reading the full text of the 15 remaining articles, 6 used only other procedures as a control (local analgesia, exercise, physiotherapy, etc.), 2 did not report available data regarding pain relief or ROM, 1 was a retrospective controlled trial, and 1 assess the efficacy of intra-articular corticosteroid injection in diabetic patients. Ultimately, 5 prospective controlled trials were included in our meta-analysis, of which 4 were RCTs^[11,18–20]^ and 1 was prospective nonrandomized trials.^[9]^

The relevant data were extracted from each eligible trials by 2 authors (MS and WW) independently. We also reviewed study design, intervention protocol, sample size, duration of follow-up, outcome measurement, and loss to follow-up. Intention-to-treat (ITT) data were used whenever possible. If the data were not available, we used the data analyzed from the available data or data from the analysis of treatment received. When the data were not reported in the original article, data were extrapolated from the accompanying illustrations. We recorded the characteristics of the 5 included trials (Table 1). The weighted kappa for agreement on eligibility between reviewers was 0.86 (95% confidence interval [CI], 0.78–0.94).

**Table 1:**
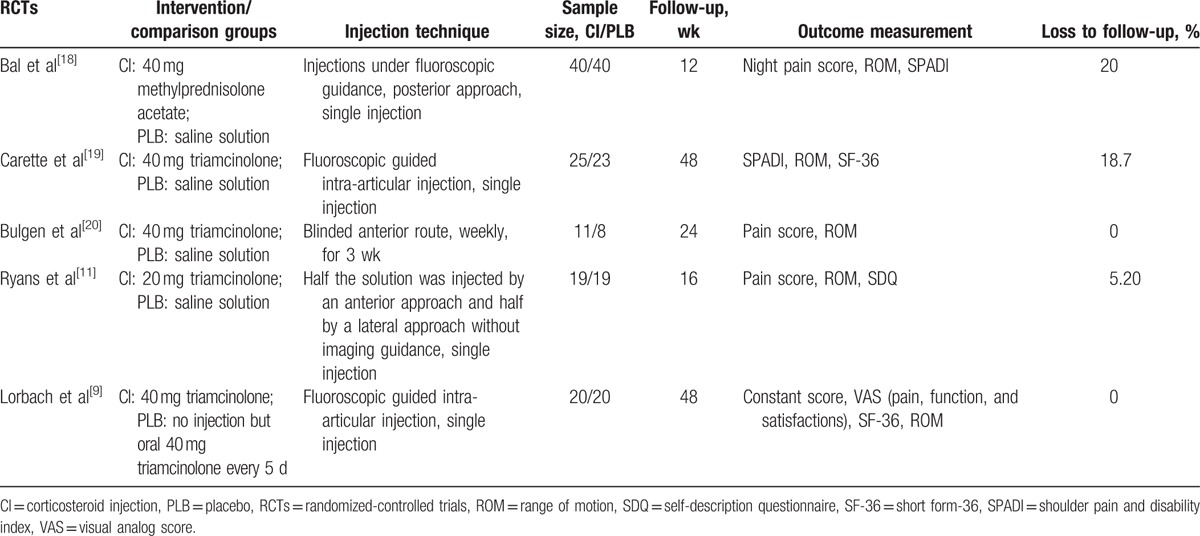
Characteristics of the included studies.

### 2.4 Methodological quality assessment

Two reviewers (SY and WW) independently assessed the methodological quality of the included trials with a 12-item scale, assessing factors such as randomization, allocation concealment, similar baseline, blinding, selective reporting, patient's compliance, loss to follow-up, similar timing, and ITT analysis,^[21]^ and we resolved disagreements through discussion (Table 2). The weighted kappa for the agreement on the trial quality between reviewers was 0.88 (95% CI, 0.78–0.99). We also used the criteria of the Grading of Recommendations Assessment, Development and Evaluation (GRADE) to evaluate the quality of the evidence.^[22]^

**Table 2:**
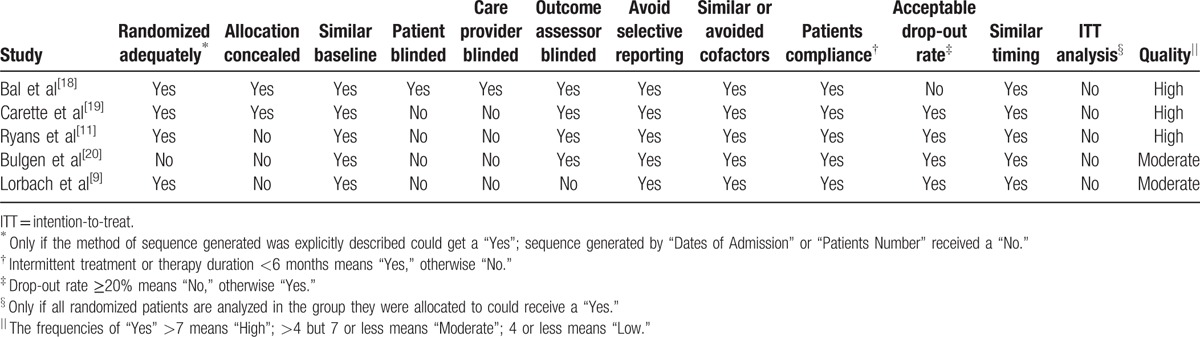
Methodological quality of the included controlled trials.

### 2.5 Outcome measures

The visual analog score (VAS) pain scores^[23,24]^ evaluated with credible measurement in the short term and long term after treatment were our primary outcome. We considered the ROM as our secondary outcome. Passive shoulder motion includes abduction, flexion, extension, and rotation (internal rotation and external rotation), and the included studies mostly used abduction, flexion, internal rotation, and external rotation, which were analyzed in our review. We defined the follow-up as a “short-term” follow-up of which duration was <8 weeks and of which more than 8 weeks but <24 as a “long-term” one.

### 2.6 Statistical analysis

The data were pooled using Review Manager 5.1.3 software (The Cochrane Collaboration, Oxford, UK). All continuous outcome measures were converted to weighted mean differences and the statistical heterogeneity was calculated with a chi-squared test on N − 1 degree of freedom (N = sample size). The inconsistency was also assessed using the formula: (Q − df)/Q × 100% (Q = the chi-squared statistic; df = degree of freedom) to describe the percentage of the variability in effect estimates attributable to the heterogeneity.^[25]^ I^2^ values of 75%, 50%, and 25% were considered as high, medium, and low heterogeneity, respectively. If there were no statistical heterogeneity among the studies, a fixed-effects model was used, otherwise, the random-effects model was used.

We did not make a funnel plots analyses because the number of studies were limited. Sensitivity analyses were performed to evaluate whether specified factors could influence the overall effects of pain scoring and ROM. We omitted the studies one by one, which is nonrandomized,^[20]^ or with small sample size and using oral medications as control treatment.^[9]^

## 3 Results

A total of 1166 eligible articles were revealed in our study, among which we rejected 1151 articles according the title and abstract. We reviewed the remained 15 studies for full papers. We further excluded 10 unsuitable articles based on our inclusive and exclusive criteria. Finally we included 4 RCTs and 1 prospective, nonrandomized, controlled trial in this meta-analysis. A flow chart is shown in Fig. 1.

### 3.1 Pain relief in short term and long term

**Figure 2:**
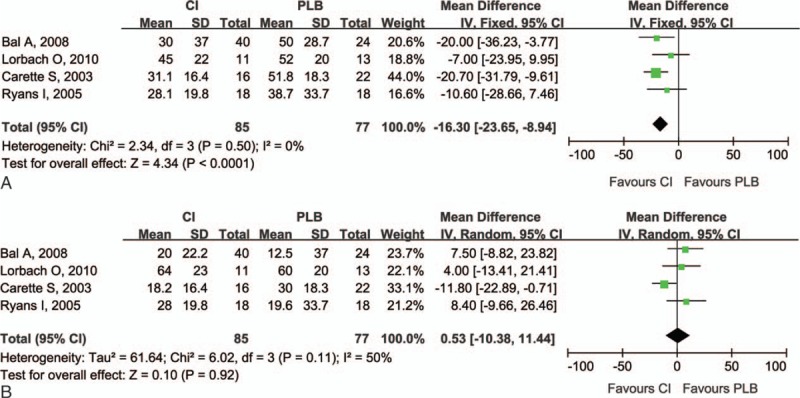
Forest plot for pain relief at (A) 0 to 8 weeks and (B) 9 to 24 weeks. Compared with control treatment, intra-articular corticosteroid injections were more effective in reducing the pain score (*P* < .0001) at 0 to 8 weeks. However, there was no difference between the injection group and the control group at 9 to 24 weeks (*P* = .92). CI = intra-articular corticosteroid injection group, PLB = placebo group.

Compared with control treatment, intra-articular corticosteroid injections were more effective in reducing the pain score (*P* < .001) at 0 to 8 weeks (Fig. 2). However, there was no difference between the injection group and the control group at 9 to 24 weeks (*P* = .92). The resulting pain scores at different times had a low heterogeneity (0–8 weeks: I^2^ = 0%; 9–24 weeks: I^2^ = 50%). The overall results of pain score analyses were not changed by omitting the trial, which consisted of a small sample size and used oral medications as the control treatment^[9]^ (Table 3).

**Table 3:**
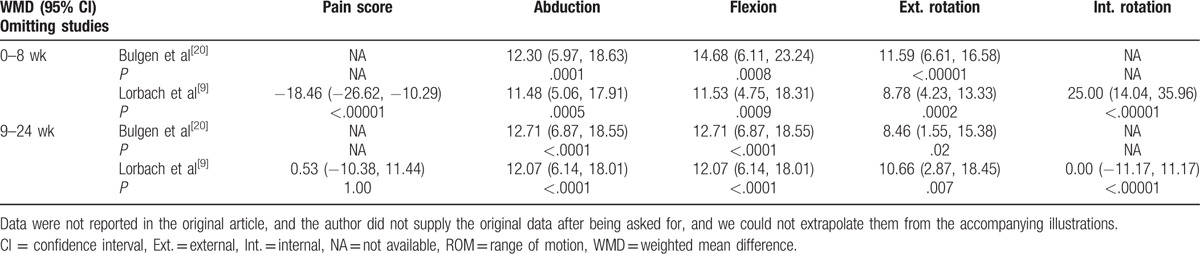
Overall results of pain score and ROM analyses by omitting the trial with small sample size or using oral-medication control.

### 3.2 ROM in short term and long term

**Figure 3:**
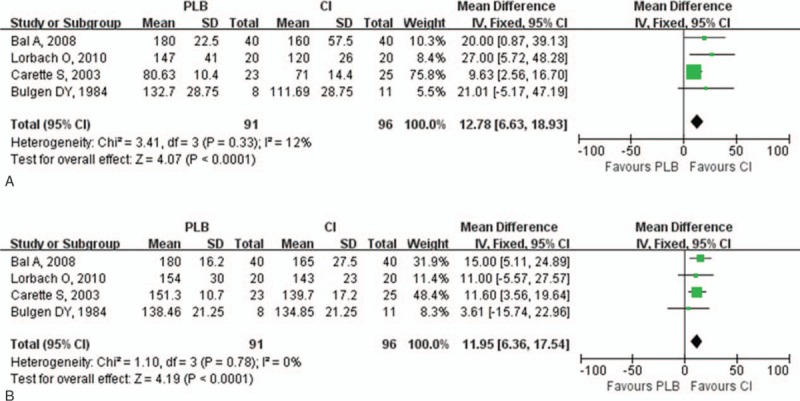
Forest plot for range of abduction at (A) 0 to 8 weeks and (B) 9 to 24 weeks. The range of abduction both at 0 to 8 and 9 to 24 weeks in the injection group was greater than in the control group (0–8 weeks: *P* < .0003; 9–24 weeks: *P* < .0001). CI = intra-articular corticosteroid injection group, PLB = placebo group.

**Figure 4:**
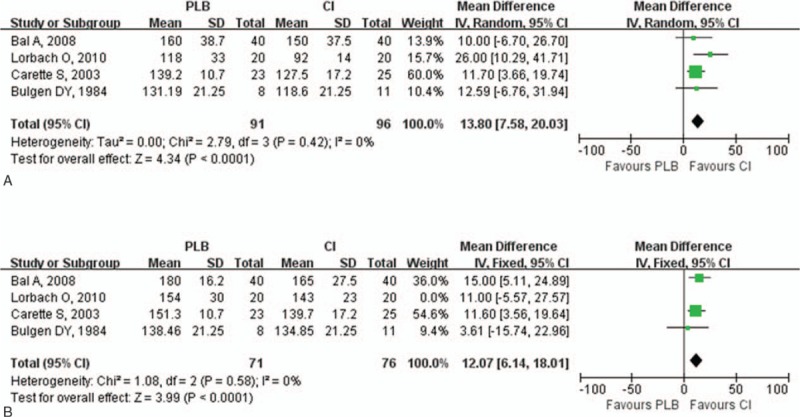
Forest plot for range of flexion at (A) 0 to 8 weeks and (B) 9 to 24 weeks. Compared with control group, there was greater improvement in the range of flexion both in the short-term and long-term follow-ups (0–8 weeks: *P* < .0001; 9–24 weeks: *P* < .0001). CI = intra-articular corticosteroid injection group, PLB = placebo group.

**Figure 5:**
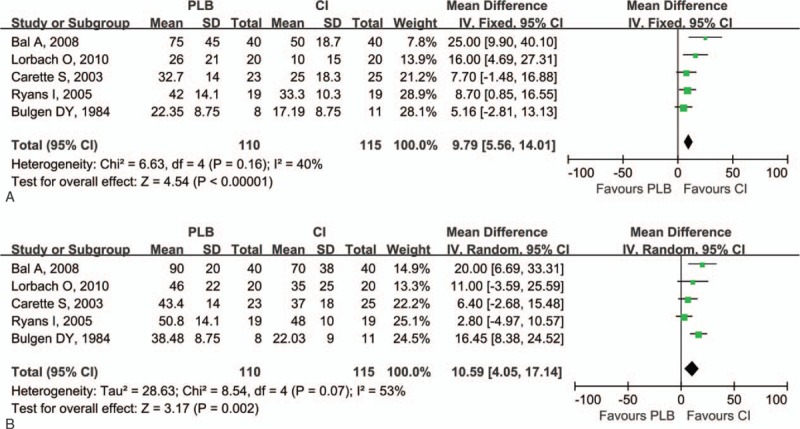
Forest plot for range of external rotation at (A) 0 to 8 weeks and (B) 9 to 24 weeks. The range of external rotation both at 0 to 8 and 9 to 24 weeks in the injection group was greater than in the control group (0–8 weeks: *P* = .0002; 9–24 weeks: *P* = .002). CI = intra-articular corticosteroid injection group, PLB = placebo group.

**Figure 6:**
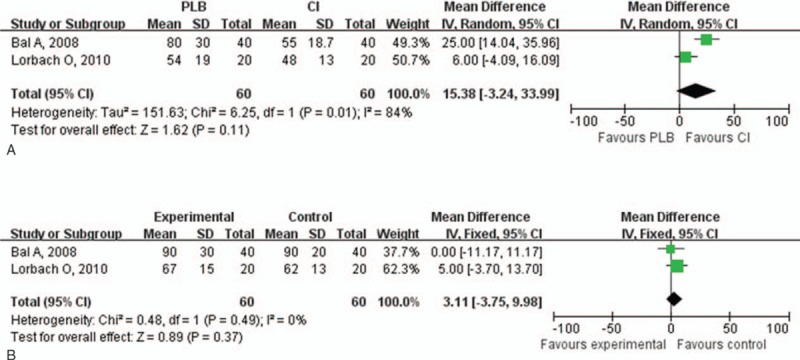
Forest plot for range of internal rotation at (A) 0 to 8 weeks and (B) 9 to 24 weeks. Compared with control group, there was greater improvement in the range of internal rotation at 0 to 8 weeks (*P* = .0001), but there was no difference at 9 to 24 weeks (*P* = .37). CI = intra-articular corticosteroid injection group, PLB = placebo group.

Improvement of ROM in the injection group was assessed by the range of abduction, flexion, external rotation, and internal rotation greater. The range of abduction both at 0 to 8 and 9 to 24 weeks in the injection group was greater than in the control group (0–8 weeks: *P* < .001; 9–24 weeks: *P* < .001; Fig. 3). Compared with the control group, there was greater improvement in the range of flexion both in the short- and long-term follow-up (0–8 weeks: *P* < .001; 9–24 weeks: *P* < .001; Fig. 4). The range of external rotation both at 0 to 8 and 9 to 24 weeks for the injection group was greater than in the control group (0–8 weeks: *P* < .001; 9–24 weeks: *P* = .002; Fig. 5). Compared with the control group, there was greater improvement in the range of internal rotation at 0 to 8 weeks (*P* < .001), but there was no difference at 9 to 24 weeks (*P* = .37; Fig. 6). Most of these analyses had no or low heterogeneity (abduction: 0–8 weeks, I^2^ = 12%; 9–24 weeks, I^2^ = 0%; flexion: 0–8 weeks, I^2^ = 0%; 9–24 weeks, I^2^ = 0%; external rotation: 0–8 weeks, I^2^ = 40%; internal rotation: 9–24 weeks, I^2^ = 0%), while the other 2 analyses resulted in high heterogeneity (external rotation: 9–24 weeks, I^2^ = 76%; internal rotation: 0–8 weeks, I^2^ = 84%). The overall results of ROM analyses were not changed by omitting the trial, which was nonrandomized,^[20]^ or consisted of a small sample size and included the use of oral medications as the control treatment.

## 4 Discussion

This literature review of existing RCT and of prospective, nonrandomized, controlled trials revealed that intra-articular corticosteroid injections resulted in improvement of clinical outcome measures of ACS based on VAS pain scores and ROM, but this improvement was different in the short-term and the long-term follow-up. At 0 to 8 weeks of follow-up, both of VAS pain scores and ROMs were improved by intra-articular corticosteroid injection when compared with the control group. However, at 9 to 24 weeks of follow-up, the ROMs had improved but there was no difference between the injection and control treatment in VAS pain scores.^[26]^

### 4.1 Limitations

Our meta-analysis has some limitations. First, due to the limited number of included trials, we could not analyze the influence of other clinically relevant factors, such as the baseline severity of shoulder pain, initial and additional medications, and dosage of corticosteroids. Second, missing information such as loss to follow-up led to incomplete data and potential bias. Third, the small sample sizes and moderate methodological qualities of some included studies^[9,20]^ might cause bias in our meta-analysis. However, by omitting these 2 trials, the results for the overall analysis of pain relief and ROMs were not changed.

A previous systematic review^[14]^ of 8 studies evaluated the effects of intra-articular corticosteroid injections on ACS. Of these, 3 studies compared the intra-articular corticosteroid injections to placebo. Our review was the first to attempt to include all the available prospective evidence comparing intra-articular corticosteroid injections and placebo. We developed explicit inclusion and exclusion criteria.

In addition, the 5 included trials were prospective controlled studies, of which 4 were RCTs. We performed a sensitivity analysis to explain the heterogeneity and to provide additional insights into the potential influential factors of the healing effect on pain relief and ROM improvement of corticosteroid injections for adhesive capsulitis.

According to the GRADE system of rating quality of evidence, most of the included trials reviewed in this meta-analysis began with moderate-quality evidence,^[24]^ but were downgraded by 5 categories of limitations (Table 4). Insufficient participants in most studies may cause limitations, and adequate sequence generation, allocation concealment, and blinding were described explicitly, which could make the limitation not serious. The main reason to downgrade the level of evidence was that the imprecision probably resulted from the small sample sizes. Thus, the present conclusion regarding the efficacy of intra-articular corticosteroid injections on adhesive capsulitis should be considered with caution.

**Table 4:**
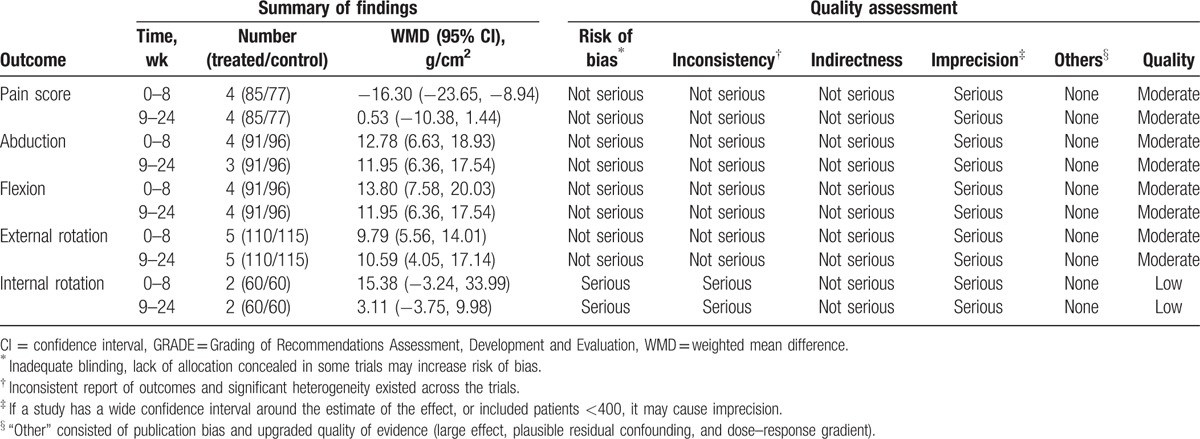
GRADE evidence profile of analyses.

Intra-articular steroid injections have been recommended in ACS based on the fact that it can diminish the inflammation that is thought to be the cause for the condition. There has been controversy regarding the ability of intra-articular corticosteroid injections to relieve pain and restore function in patients with adhesive capsulitis in the short term and the long term. In our meta-analysis, intra-articular corticosteroid injections were more effective in pain relief in the short term, but this pain relief did not sustain in the long term. In addition, intra-articular corticosteroid injections resulted in greater improvement in passive ROM both in the short and the long terms. However, the duration of follow-up was short after treatment in this study (maximum, 24 weeks). The natural history of adhesive capsulitis shows that most patients achieve the maximal outcome between 2 and 4 years after treatment. On the other hand, some studies have demonstrated severe loss of shoulder motion, mild residual pain, disability of activities of daily living, and weakness at longer-term follow-up (as long as 7 years).^[27–30]^ With a good knowledge of pathogenesis, it will become easy to determine the treatment modalities that could improve objective and subjective measures in the disease course. Therefore, it was reasonable to research the inclusion of studies with fewer than 2 years of follow-up.

Numerous authors have reported that complete resolution is not inevitable.^[28,30]^ In a well-conducted study of 62 patients, 50% of the patients continued to have either mild pain or stiffness of the shoulder after 7 years.^[30]^ According to our analysis, the use of intra-articular corticosteroid injections, significantly improved ROM in the corticosteroid injection group, both in the short term and long term, which indicates that intra-articular corticosteroid injections are beneficial for the patients’ functional recovery. In addition, intra-articular corticosteroid injections resulted in fast pain relief. However, the pain relief did not last in the long term. These data suggested that intra-articular corticosteroid injections are helpful for pain relief in only the short term.

## 5 Conclusions

In summary, intra-articular corticosteroid injections were more effective in pain relief in the short term. Unfortunately, this pain relief did not last in the long term. Intra-articular corticosteroid injections resulted in greater improvement of passive ROM both in the short and the long terms.

## Acknowledgment

The authors wish to thank all the corresponding authors from the included trials for their kind assistance in obtaining additional data that contributed to our meta-analysis.
